# Persistent innate immune dysfunction and ZIKV replication in the gastrointestinal tract during SIV infection in pigtail macaques

**DOI:** 10.3389/fimmu.2025.1535807

**Published:** 2025-03-04

**Authors:** Jennifer Tisoncik-Go, Thomas B. Lewis, Leanne S. Whitmore, Kathleen Voss, Skyler Niemeyer, Jin Dai, Paul Kim, Kai Hubbell, Naoto Iwayama, Chul Ahrens, Solomon Wangari, Robert Murnane, Paul T. Edlefsen, Kathryn A. Guerriero, Michael Gale, Deborah H. Fuller, Megan A. O’Connor

**Affiliations:** ^1^ Washington National Primate Research Center, University of Washington, Seattle, WA, United States; ^2^ Department of Immunology, University of Washington, Seattle, WA, United States; ^3^ Center for Innate Immunity and Immune Disease (CIIID), University of Washington, Seattle, WA, United States; ^4^ Department of Microbiology, University of Washington, Seattle, WA, United States; ^5^ Biostatistics Bioinformatics and Epidemiology (BBE), Program of the Vaccine and Infectious Disease (ViDD) Division, Fred Hutchinson Cancer Center, Seattle, WA, United States; ^6^ Department of Microbiology and Immunology, Institute on Infectious Diseases, University of Minnesota, Minneapolis, MN, United States

**Keywords:** Zika virus, co-infection, simian immunodeficiency virus (SIV), nonhuman primate, innate immunity

## Abstract

Mosquito-borne flaviviruses, including dengue (DENV) and Zika (ZIKV) viruses, have caused widespread epidemics in areas with high HIV prevalence, partly due to the expanded geographic range of arthropod vectors. Despite the occurrence of large flavivirus outbreaks in areas with high HIV prevalence, little is known about the effects of flavivirus infection in people living with HIV (PLWH). Here, we use a pigtail macaque model of HIV/AIDS to investigate the impact of simian immunodeficiency virus (SIV)-induced immunosuppression on ZIKV replication and pathogenesis. During acute SIV infection, peripheral ZIKV cellular targets expanded and innate immune activation increased. *In vitro*, peripheral blood mononuclear cells (PBMC) from SIV infected pigtail macaques were less permissive to ZIKV infection. *In vivo*, ZIKV viremia was delayed and ZIKV was more persistent in the gastrointestinal tissues of SIV-ZIKV co-infected animals. This persistence was associated with changes in innate cellular (monocytes, neutrophils) recruitment to the blood and tissues, reduced anti-ZIKV immunity, and sustained expression of peripheral inflammatory and innate immune genes. Collectively, these findings uniquely suggest that untreated SIV infection may promote inflammatory cellular innate responses and create a state of persistent immune activation that contributes to prolonged ZIKV viremia and persistence in the gastrointestinal tract. Furthermore, these results suggest that PLWH and other immunocompromised individuals could be at higher risk for prolonged ZIKV infection, potentially extending the window of ZIKV transmission. These insights highlight the importance of including PLWH in strategies for deploying vaccines and treatments against ZIKV.

## 1 Introduction

Vector-borne diseases account for greater than 17% of global infectious diseases and in the past decade flavivirus outbreaks, including dengue (DENV), yellow fever (YFV) and Zika (ZIKV) have raised significant public health concerns (1). There is also an ongoing threat of esoteric flaviviruses including spondweni, usutu, ilheus, rocio, or wesselsbron, which have epidemic potential (2). While most flavivirus infections are asymptomatic and self-limiting, severe forms of disease, including hemorrhagic, encephalitic, or congenital disease, or death can occur (2). The factors associated with severe flavivirus disease and neurological manifestations are not well understood (3, 4). However, certain groups are more vulnerable and face an increased risk for severe disease, including children, the elderly, pregnant women, and immunocompromised individuals (such as people living with HIV (PLWH)) (2, 5). Flaviviruses are endemic in over 100 countries, many of which have high rates of HIV infection. Africa accounts for 90% of global YFV cases and it is estimated that more than 48 million people in Africa are infected with Dengue every year (6). Despite the frequent outbreaks and overlap of these infections in high HIV prevalence regions, there is still limited knowledge regarding flavivirus pathogenesis in the context of HIV infection. Misdiagnosis due to symptom overlap with other flaviviruses or incorrect identification of etiologic agents from outbreak cases using PCR or serological methods further complicates the accurate assessment of flavivirus burden (6). Studies on flavivirus infection and disease outcomes often exclude PLWH and this limits our understanding of how HIV co-infection affects disease outcomes. Currently, highly effective vaccines against DENV, WNV, and ZIKV do not exist. While a live-attenuated YFV vaccine 17D is available, it is contraindicated in infants and in immunosuppressed individuals and is relatively contraindicated in the elderly, pregnant women, and PLWH due to poor immunogenicity or severe adverse reactions (7–10). Therefore, there is a need to better understand flavivirus pathogenesis in vulnerable and at risk populations and the factors associated with severe flaviviral outcomes.

The 2015-2016 Zika outbreak in the Americas led to over 500,000 infections and ZIKV has since become endemic in the region, with more than 40,000 cases reported in 2024 (11). ZIKV is primarily transmitted through mosquito bite, but it can also spread through sexual contact or from mother to fetus (12–14). In most cases, ZIKV is mild and self-limiting, but it can cause severe neurological disorders such as Guillain-Barré syndrome (GBS) in adults and congenital Zika syndrome (CZS) in newborns exposed *in utero* (15, 16). CZS is characterized by severe defects in cranial morphology, ocular abnormalities, muscle contractures and neurological impairments (17). Nonhuman (NHP) models of ZIKV infection have been instrumental in studying the disease, as they replicate human infection routes, mild disease progression, and vertical transmission to the developing fetus (18–22). In NHP models, fetal loss occurred in 26% of ZIKV exposed animals, suggesting fetal loss in asymptomatic pregnant women may be underreported (20). Moreover, altered myelination in normocephalic fetuses following maternal-to-fetal ZIKV transmission argues ZIKV infection *in utero* can impact pre- and post-natal neurologic development (23). These findings highlight the NHP model as a valuable tool for understanding ZIKV pathogenesis and for testing vaccines.

Persistent immune exhaustion and activation during HIV infection, even under potent antiretroviral drug therapy (ART), contributes to increased morbidity and mortality. Some of these biomarkers exist despite high CD4 T-cell counts and/or early ART treatment and do not preclude individuals from inflammation-associated co-morbidities (24). In particular, elevated levels of type I interferons (IFNs) and persistent activation of interferon stimulated genes (ISGs) can lead to tolerance to ISG functions and attenuate the anti-viral type I IFN response (25). Thus, weakened responsiveness to type I IFN signaling during HIV infection can impair pathogen clearance of subsequent co-infections.

Both innate and adaptive immune responses are important for clearing ZIKV and preventing re-infection (26, 27). We and others have shown that circulating monocytes and dendritic cells are the primary cellular blood targets of ZIKV infection in humans and NHP (28–30). These cells contribute to disease pathogenesis through the production of inflammatory mediators, but also play a key role in the antiviral type I interferon (IFN) response (31). During HIV infection, the frequency of blood monocyte frequencies increase, and while ART reduces overall monocyte frequencies, inflammatory monocytes remain elevated (32). ZIKV can persist in bodily fluids and tissues for weeks to months, though the mechanisms behind this persistence are not well understood (14, 33). Previous research in the NHP HIV/AIDS model has shown that the early type I IFN response and impaired humoral responses during simian immunodeficiency virus (SIV) infection may delay ZIKV clearance from blood and lymphoid tissues (34). However, several questions remain regarding the impact of HIV on ZIKV infection, including: 1) Does SIV infection alter the permissiveness of cells to ZIKV?, 2) How does SIV infection influence ZIKV persistence in tissues like the gastrointestinal tract or central nervous system?, 3) Do innate immune responses during acute or post-acute phases differ during SIV-ZIKV co-infection and ZIKV infection alone? To address these questions, we utilized the pigtail macaque (PTM) model of HIV/AIDS. This species is ideal for studying HIV-induced immunosuppression and co-infections due to its inherent mucosal dysfunction, which accelerates AIDS progression (35–37). By examining longitudinal innate immune responses in the blood, gastrointestinal and lymphoid tissues, we aimed to better understand the impact of SIV infection on ZIKV viral persistence. Here, we investigate how SIV infection affects the susceptibility of peripheral blood mononuclear cells (PBMC) to ZIKV infection, ZIKV persistence and the immune response.

## 2 Materials and methods

### 2.1 Study design and animal welfare

A total of 14 pigtail macaques (male/female, aged 4-11 years, 6-13 kg) were used. **Supplementary Table 1** details animal characteristics, including MHC haplotypes and experimental vaccination history. Prior to enrollment, all animals were pre-screened and confirmed seronegative for the presence of antibodies to West Nile, dengue, and Zika viruses. At least 2 months prior to enrollment into the study, eight animals were previously enrolled in studies in which they received an experimental hepatitis B virus (HBV) vaccine consisting of a combination of CD180 targeted DNA and recombinant protein vaccines comprised of HBV core and surface antigens (38) and/or a replicating RNA COVID-19 vaccine (39) (**Supplementary Table 1**) and were evenly distributed between the control and experimental groups. Seven pigtail macaques were infected intravenously with 10,000 infectious units (I.U.) of SIVmac239M (40) (gift from Dr. Brandon Keele, AIDS and Cancer Virus Program, Frederick National Laboratory for Cancer Research) and then co-infected with ZIKV 9 weeks later. All animals were inoculated subcutaneously with 5 x 10^5^ PFU of the Brazil_2015_MG strain of ZIKV (GenBank: KX811222.1), as previously described (30). All animals were subject to a Simian AIDS monitoring protocol as defined by the WaNPRC guidelines (41). ZIKV RNA was not detected in any specimen tested at any timepoint in one animal in the SIV-infected group (Z14109); therefore, this animal was excluded from all post-ZIKV analysis. Animals were observed daily and full physical exams were conducted at each experimental timepoint, as previously described (30). All animals were euthanized at the study endpoint at 4 weeks post-ZIKV infection under deep anesthesia, in accordance with the 2007 American Veterinary Medical Association Guidelines on Euthanasia, by administration of Euthasol^®^ (Virbac Corp., Houston, TX).

### 2.2 Simian AIDS measurements

SIV plasma viremia was evaluated by quantitative real time reverse transcription polymerase chain reaction (RT-PCR) by the Virology and Immunology Core at the WaNPRC, as previously described (41), and by the NIAID DAIDS Nonhuman Primate Core Virology Laboratory (NHPCVL) for AIDS Vaccine Research and Development Contract. Complete blood counts (CBC) and serum chemistries were performed by the Research Testing Service (RTS) at the University of Washington Department of Laboratory Medicine. Peripheral CD4 counts were determined from CBC using flow cytometry-based methods by the Virology and Immunology Core at the WaNPRC, as previously described (42).

### 2.3 Cell culture and virus stock

Peripheral blood mononuclear cells (PBMC) were isolated from NHP whole blood collected pre-SIV inoculation (Wk-3) and at weeks 2 and 6 post-SIV inoculation, as previously described (30). PBMC were maintained in RPMI medium supplemented with 10% fetal bovine serum (FBS; HyClone), 2 mM L-glutamine, 5 mM sodium pyruvate, 1x Antibiotic Antimycotic Solution, and 10 mM HEPES (cRPMI; complete RPMI). RPMI medium used for the ZIKV inoculation was supplemented with 1% FBS, 2 mM L-glutamine, 5 mM sodium pyruvate, 1x Antibiotic Antimycotic Solution, and 10 mM HEPES (iRPMI; infection RPMI). Vero cells (WHO, Geneva, Switzerland) were cultured in complete Dulbecco’s modified Eagle medium (cDMEM) supplemented with 10% FBS, 2 mM L-glutamine, 5 mM sodium pyruvate, 1x Antibiotic Antimycotic Solution, 10 mM HEPES and 1X non-essential amino acids. Vero cells tested negative for mycoplasma contamination. All cells were maintained in a 37°C incubator with 5% CO_2_. Brazil Zika virus stock (GenBank: KX811222.1) was used for the PBMC inoculation.

### 2.4 ZIKV infection of PBMC

Following overnight incubation, PBMC cell suspensions were prepared, and the cell concentration and viability measured using the Countess 3 Automated Cell Counter (ThermoFisher Scientific). Approximately 6 x 10^6^ PBMC were inoculated with ZIKV at an MOI of 2 in a total volume of 200 µL RPMI infection medium (iRPMI) at 37°C for 2 hours (h). Cells were gently mixed by pipetting at 20 minute (min) intervals during incubation. After 2 h, the cells were spun at 300 relative centrifugal force (rcf) for 3 min at room temperature and the inoculum carefully removed without disturbing the cell pellet. The cells were washed with 300 µL iRPMI and then resuspended in pre-warmed complete RPMI (cRPMI). A total of 5 x 10^5^ PBMC were added to each well of a 24-well plate containing 1 mL of cRPMI. The plates were returned to 37°C and incubated until the designated timepoint for sample collection. At 4, 24, and 48 h post-ZIKV inoculation, supernatants were collected and spun at 300 rcf for 3 min at 4°C. 100 µL Versene solution (ThermoFisher Scientific) was added to each well to dislodge adherent cells from the TC plate. The clarified supernatant was transferred to a new tube and banked at -80°C until further processing by plaque assay or qRT-PCR assay. The Versene solution containing PBMC was added to the PMBC cell pellet from the supernatant and then spun at 300 rcf for 3 min at 4°C. The supernatant was carefully removed from the cell pellet and discarded. The cell pellet was then resuspended in 700 µL QIAzol for RNA analysis.

### 2.5 Plaque assay

Vero cells (WHO, Geneva, Switzerland) were seeded at a density of 5 x 10^5^ cells per well in 6-well plates. The next day, the medium was removed from the monolayers and 200 µL of 10-fold serial dilutions of virus-containing supernatant in DMEM containing 2% FBS was added to respective wells in duplicate. Vero cell monolayers were incubated at 37°C for 2 h, with rocking at 15 min intervals. Monolayers were overlaid with 1% low-melting point SeaPlaque^®^ agarose (Lonza), set at 4°C for at least 20 min, and then returned to the 37°C incubator. Plaques were visualized and counted 4 days later by crystal violet staining.

### 2.6 NanoString nCounter assay and gene analysis

The NanoString nCounter platform (NanoString, Seattle, WA, USA) was used to quantify mRNA counts in PBMC processed from whole blood at pre- and post-SIV and ZIKV infection timepoints. RNA was isolated from cryopreserved PBMC samples collected at pre-SIV inoculation (Wk -3), 2 and 6 weeks post-SIV using the miRNeasy Mini Kit (QIAGEN). RNA was isolated from 1-2 x 10^6^ PBMC resuspended in 700 µl QIAzol collected at week 7/Day -14 pre-ZIKV inoculation, and at days 2, 4, 7, 10, 14 and 21 post-ZIKV challenge using the miRNeasy Micro Kit (QIAGEN). From each sample, 100 ng RNA was loaded in accordance with manufacturer’s instructions for targeted expression with 2 custom-built curated NanoString Human Panels of 44 and 60 genes of interest which both represent gene biomarkers of innate immune activation and response, interferon response, and inflammatory response. Due to the small number of genes represented on the Code Set, nCounter data normalization was performed using a method which calculates a ratio between genes of interest to housekeeping genes (43). The ratio is calculated by dividing the counts of the genes of interest by the geometric mean of 4 housekeeping genes which have the lowest coefficient of variance across all samples. This is done for each sample independently, which generates normalized expression for the genes of interest. Significant differences (nominal *P*-val <0.01) were determined between baseline and post-infection time points for each group and between groups at each time point.

### 2.7 Histology

At necropsy representative samples of all tissues and organs were collected in formalin and after fixation were paraffin embedded and sectioned at 3-5 μm. For basic histology, sections were stained with hematoxylin and eosin. All histological findings are summarized in **Supplementary Table 2**.

### 2.8 Immunophenotyping

Isolated PBMC, rectal and peripheral lymph node biopsy cells were assessed for viability with a live/dead stain (Life Technologies) and stained with a panel of antibodies, details described in **Supplementary Table 3**, in brilliant stain buffer (BD Biosciences) to identify innate immune cells as previously described (30) and were gated according to **Supplementary Figure 1**. Paraformaldehyde fixed cells were acquired on a LSRII (BD Biosciences) using FACS Diva software (version 8). Samples were analyzed using FlowJo software version 10.8.1 (FlowJo, LLC). All events were first gated on FSC singlets, CD45^+^ leukocytes, live, and then cells according to FSC-A and SSC-A profiles. Immune cells were identified as follows: plasmacytoid dendritic cells (DCs) (CD20^-^CD3^-^HLA-DR^+^CD14^-^CD123^+^CD11c^-^), myeloid DCs (CD20^-^CD3^-^HLA-DR^+^CD14^-^ CD123^-^CD11c^+^), monocytes (CD20^-^CD3^-^HLA-DR^+^CD14^+^CD16^+/-^), and neutrophils (CD3^-^CD11b^+^CD14^+^SSC-A^Hi^). AXL receptor tyrosine kinase (AXL) positive cells were identified after FMO subtraction and meeting a cellular threshold (>100 cells/gate).

### 2.9 Multiplex bioassay

Cytokine and chemokine levels in plasma and cerebrospinal fluid (CSF) were analyzed using a custom nonhuman primate ProcartaPlex 24-plex immunoassay (ThermoFisher Scientific), per the manufacturer’s protocol. The levels of the analytes were assessed on a Bio-Plex 200 system (Bio-Rad) and analyzed per the manufacturer’s protocol.

### 2.10 ZIKV RNA quantitation

Viral RNA load was assessed in plasma, CSF, rectal cytobrush supernatant, and tissues using a ZIKV-specific RT-qPCR assay, as previously described (30). RNA was isolated from plasma and rectal cytobrush supernatant collected pre-challenge and at 1, 2, 3, 4, 7, 10, 14 and 21 days post-infection (dpi) and at necropsy (24-28 dpi). RNA was isolated from CSF collected pre-challenge and at days 4, 7 and 21 post-challenge and at necropsy (24-28 dpi). Rectal and PLN biopsy tissues were collected pre-challenge and at days 7 and 21 post-challenge and lymphoid and gut tissues collected at necropsy (24-28 dpi). The iScript Select cDNA Synthesis Kit (Bio-Rad) was used for gene-specific cDNA synthesis and cDNAs were quantified on a QuantStudio Real-Time PCR System (ThermoFisher Scientific). Ct values <39 in at least 2 of the triplicates and falling within the standard curve determined from diluted known quantities of ZIKV genome were considered positive.

### 2.11 Measurement of gut integrity and neuroinflammation

Plasma and/or CSF quantification by ELISA of human soluble CD14 (sCD14), human fatty acid binding protein 2 (FABP2) (Fisher Scientific, Waltham, MA) or human LPS binding protein (LBP) (Biometec, Germany) was performed per the manufacturer’s instruction. Plasma was diluted as follows: 1:200 (sCD14), 1:2 (FABP2), or 1:3 (LBP). CSF was diluted 1:5 (sCD14). Results were analyzed using Prism version 10.4.1 (GraphPad) and using a four- or five- parameter logistic (4- or 5-PL) function for fitting standard curves.

### 2.12 Anti-ZIKV IgG quantification

NHP sera and/or plasma samples were assessed for anti-ZIKV envelope (E) IgG binding titers by an Enzyme-Linked Immunosorbent Assay (ELISA). Purified NHP IgG (MyBioSource MBS539659) was serially diluted to establish a range of IgG standards. ZIKV E protein (Fitzgerald Industries International, 30-1932) was diluted to 0.5 μg/mL and was used as the capture antigen. Capture antigen and IgG standards were coated overnight on high-binding 96-well plates (Costar 3590) to produce test and standard wells, respectively. All wells were subsequently blocked in blocking buffer (5% w/v nonfat dried milk (Bio-Rad Laboratories 1706404) and 0.05% v/v Tween-20 in PBS). Samples were diluted 1:100, 1:200, and/or 1:400 and tested in triplicate. NHP IgG standard and test wells were probed by a goat anti-monkey IgG antibody conjugated to Horseradish peroxidase (HRP) (Abcam ab112767). SureBlue Reserve TMB substrate (KPL) was added to all wells to initiate a color change reaction catalyzed by HRP. Reaction was stopped after 30 minutes with 1N HCl (VWR) and absorbance at 450nm (Abs_450_) was measured on an EMax plate reader (Molecular Devices). Abs_450_ of standard wells were used to produce a 5PL logistic fit (GraphPad Prism). Abs_450_ of test wells were converted to μg/mL of anti-ZIKV E IgG binding titers via the 5PL logistic fit.

### 2.13 Plaque reduction neutralization test

NHP sera collected pre-challenge (Day -14) and at necropsy (24-28 dpi) were tested in PRNT assay for neutralizing antibody production, as previously described (44). The PRNT assay was performed using serial two-fold dilutions of the serum samples. The highest serum dilution reducing plaque numbers by 50% (PRNT_50_) were determined with a limit of detection (LOD) of 1:50. The assay was repeated twice in triplicate using the ZIKV Brazil 2015 virus.

### 2.14 Statistical analysis

Non-parametric statistical methods were employed for all comparisons, unless otherwise noted. Specifically, Kruskal-Wallis tests were used for comparisons across timepoints in *in vitro* experiments, paired Wilcoxon tests were used to evaluate cell fraction differences to baseline at each timepoint, and Mann-Whitney tests were used to compare continuous values across groups. All analyses were conducted using two-sided tests at the 0.05 level. Analyses were conducted in Prism version 10.4.1 (GraphPad). Significant differences (nominal *P*-val <0.01) in gene expression were determined using a t test that compared baseline and infection timepoints for each group and between groups at each timepoints.

## 3 Results

### 3.1 *In vitro* ZIKV replication is impaired in PBMC from NHP acutely infected with SIV

**Figure 1:**
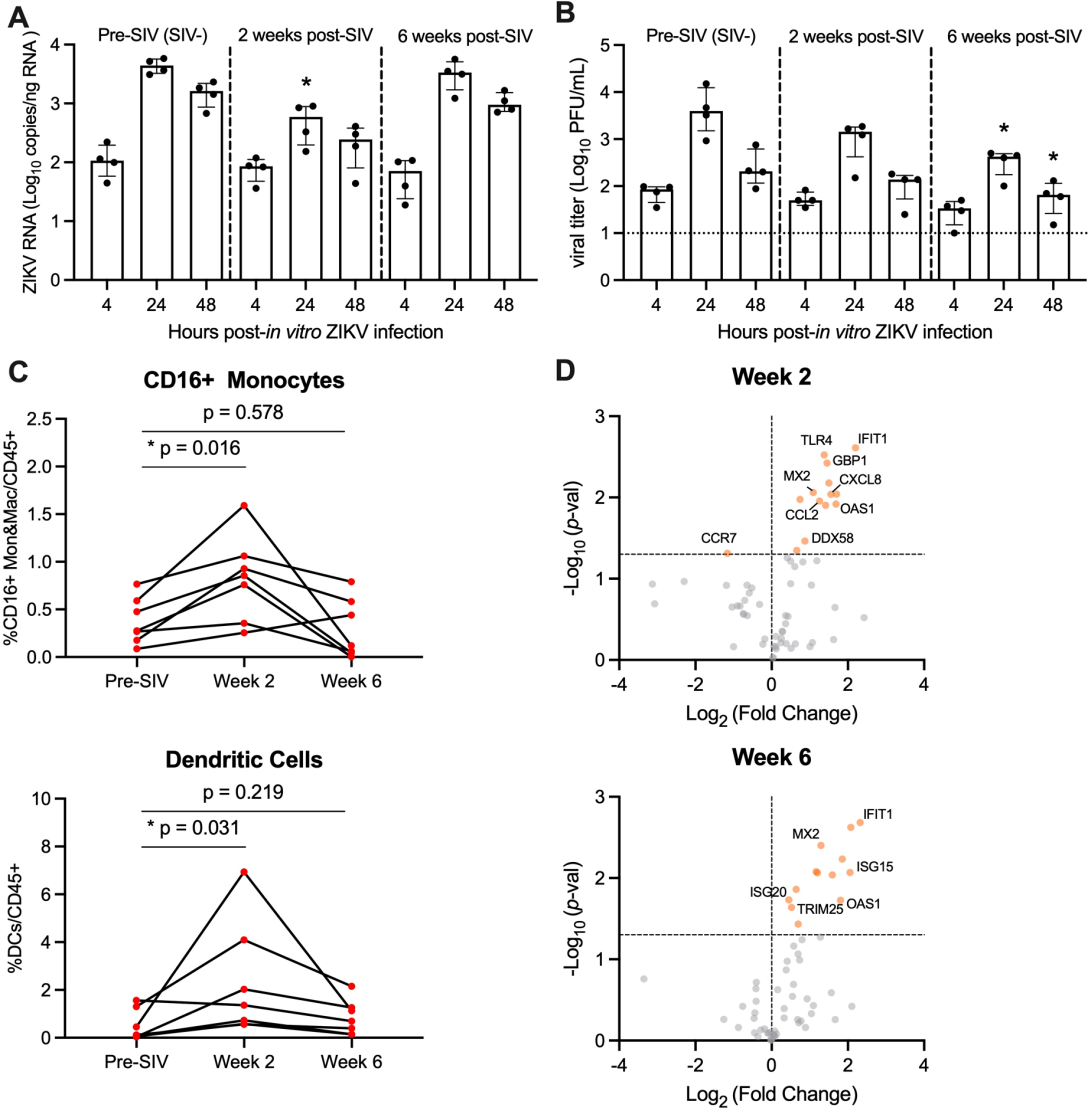
PBMC from SIV-infected PTM are less permissive to *in vitro* ZIKV infection. **(A, B)** Peripheral blood mononuclear cells (PBMC) were isolated from pigtail macaques prior to and at 2 and 6 weeks post-SIV infection and infected *in vitro* with ZIKV Brazil at a multiplicity of infection (MOI) of 2. Cells and supernatant were harvested 4, 24, and 48 hours post-infection. **(A)** Quantitative real-time PCR (qRT-PCR) for ZIKV RNA in PBMC. **(B)** Plaque assay for infectious Zika virus. **(A, B)** Medians with interquartile ranges are shown. Kruskal-Wallis test versus pre-SIV levels, p-values * ≤ 0.05. **(C)** Frequency of CD16+CD14+ monocytes and macrophages (Mon&Mac) (top panel) and dendritic cells (lower panel) in blood from uninfected and SIV-infected pigtail macaques. Wilcoxon matched-pairs signed rank test, p-values ≤ 0.05 considered significant. **(D)** Gene expression of PBMC in blood at Week 2 post-SIV (top panel) and Week 6 post-SIV (bottom panel). t-test between each timepoint relative to pre-SIV, p-values *<0.01 shown by orange dots.

Pigtail macaques (PTM) (n=7) were infected with SIVmac239M and blood was collected prior to and at 2 and 6 weeks post-SIV infection. Both post-infection timepoints are associated with significant declines in peripheral CD4 counts and correspond with SIV peak and viral setpoint, respectively (**Supplementary Figure 2**). To evaluate whether SIV infection alters the permissivity of peripheral blood mononuclear cells (PBMC) to Zika virus (ZIKV) infection, we isolated fresh PBMC from SIV+ PTM (n=4) at weeks 2 and 6 post-SIV infection and inoculated cells with ZIKV *ex vivo*. PBMC isolated prior to SIV infection (pre-SIV, SIV-) served as a control. At 4, 24 and 48 hours post-ZIKV infection (hpi), cells and culture supernatants were collected to measure ZIKV RNA and viral titer. As expected, control pre-SIV PBMC were permissive to ZIKV infection, as measured by qRT-PCR and plaque assay, with peak viral replication at 24 hpi (**Figures 1A, B**, **Supplementary Figure 3**). ZIKV RNA levels in SIV+ PBMC collected at week 2 post-SIV were significantly decreased at 24 hpi compared to pre-SIV PBMC while SIV+ PBMC collected at week 6 post-SIV had similar ZIKV RNA levels to that in pre-SIV PBMC (**Figure 1A**). The kinetics of ZIKV were similar in pre-SIV and SIV+ PBMC; however, significantly lower levels of infectious virus were observed at 24 and 48 hpi in SIV+ PBMC collected at week 6 post-SIV (**Figure 1B**; **Supplementary Figure 3**). Supernatants from ZIKV-infected PBMC cultures were subjected to multiplex immunoassay to measure cytokine and chemokine concentration changes at 4, 24, and 48 hpi. All cultures accumulated the pro-inflammatory cytokines MCP-1 and VEGF-A during the 48 hr post-infection period; however, there was only a trend for an increase of IL-5 (p = 0.061) in Week 2 SIV-infected cultures relative to pre-SIV at 24 hr (**Supplementary Figure 4**). These data suggest that cells from acutely SIV-infected animals are less permissive to ZIKV infection.

### 3.2 Expansion of ZIKV cellular targets in the blood during acute SIV infection

Monocyte frequencies increase in the blood during HIV and SIV infection and are the primary targets of ZIKV infection (28–30, 32). Since ZIKV replication was lower in PBMC derived from SIV-infected animals, we next determined whether this was because of fewer ZIKV cellular targets. To test this, we evaluated innate cells in fresh blood from SIV+ and SIV- PTM by flow cytometry. During acute SIV infection, the frequency of CD16+ monocytes and dendritic cells (DCs) significantly increased at Week 2, with levels returning to pre-infection levels at Week 6 (**Figure 1C**). We next examined AXL expression on CD16+ monocytes and DCs in response to SIV infection, as the TAM receptor tyrosine kinase is a known ZIKV viral entry receptor (45–48). *Ex vivo* AXL expression on CD16+ monocytes and DCs was unchanged at Weeks 2 or 6 post SIV-infection (**Supplementary Figure 5**). Thus, PBMC from SIV-infected PTM have similar/greater levels of ZIKV cellular targets in comparison to naïve PTM and these cells express similar levels of surface AXL, but they are less vulnerable to ZIKV infection *ex vivo*.

We next hypothesized that anti-viral responses induced early within SIV infection could influence ZIKV permissibility. To test this, we used a targeted custom-built NanoString nCounter gene expression assay to investigate a panel of 84 immune-related genes in PBMC collected at pre-SIV and at Weeks 2 and 6 post-SIV infection. Differential gene expression analysis was performed for each timepoint post-SIV infection relative to pre-SIV PBMC and identified several significantly upregulated innate immune and interferon stimulated genes (ISGs) (**Figure 1D**; **Supplementary Table 4**). With SIV infection, several genes related to innate immunity were upregulated in expression compared to pre-SIV PBMC. Notably, *IFIT1*, *MX2*, and *OAS1* ISGs were increased in expression at both Weeks 2 and 6 post-SIV. *ISG20* and *ISG15* were significantly upregulated in expression at Week 6, while *CXCL8* and *CCL2* encoding monocyte chemoattractant proteins were significantly upregulated at Week 2 post-SIV. Retinoic acid-inducible gene-I (RIG-I) signaling activated by viral infection induces the expression of these antiviral genes that, in turn, are known to restrict ZIKV replication (49, 50). These data indicate that despite the presence and expansion of ZIKV cellular targets during acute SIV infection, increased innate immune gene expression in PBMC could render monocytes refractory to ZIKV infection.

### 3.3 ZIKV co-infection does not significantly impact peripheral SIV disease progression

**Figure 2:**
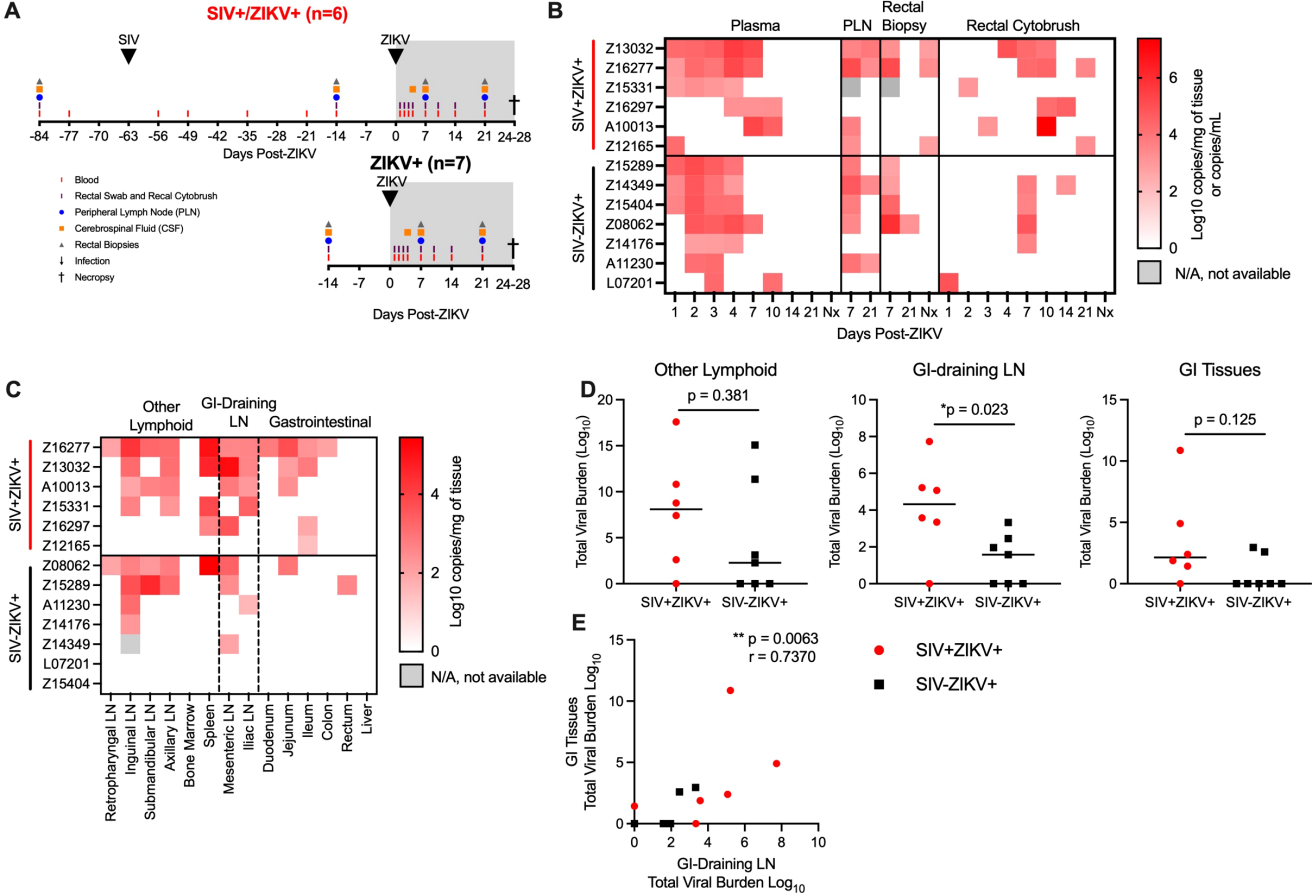
ZIKV viremia is delayed and protracted and ZIKV viral burden more persistent in gastrointestinal tissues in SIV-infected macaques. **(A)** Study design of longitudinal blood and tissue sampling following SIV and ZIKV infections in pigtail macaques. Initially n=7/group were infected with ZIKV; however, 1 animal in the SIV+ group (Z14109) displayed no evidence of ZIKV replication and thus was excluded from all post-ZIKV analysis. **(B)** Quantitative real-time PCR (qRT-PCR) for ZIKV RNA in longitudinal samples from plasma, peripheral lymph node (PLN), rectal biopsies, and rectal cytobrush until necropsy (Nx). Virus was not detected in any longitudinal cerebrospinal fluid (CSF). **(C)** ZIKV RNA in tissues collected at necropsy 24-28 DPI. Virus was not detected in brain tissue (brainstem, hippocampus, frontal lobe, parietal lobe, and occipital lobe) of any animal. Gastrointestinal (GI), lymph node (LN). **(D)** Total viral burden in other lymphoid, GI-training and GI tissues, as described in panel **(C)**. Each point represents an individual animal and/or tissue and medians are shown. Mann-Whitney test between groups, *p-values ≤ 0.05 are considered significant. **(E)** Scatter plot and Spearman’s correlation analysis of the relationship between the total viral burden in GI-draining lymph nodes and gastrointestinal tissues at necropsy. Spearman’s correlation test p-values are indicated: **p ≤ 0.01.

SIV-infected PTM were co-infected with ZIKV at 63 days (9 weeks) post-SIV infection (SIV+ZIKV+) and compared to SIV-naïve PTM infected with ZIKV (SIV-ZIKV+) (**Figure 2A**). This timepoint from the post-acute phase of SIV infection was selected as it corresponds with the establishment of viral setpoint (median SIV viremia 5.41 (1.63-6.18) log_10_ copies/mL of plasma) and there is evidence of immunosuppression including lowered, yet stable peripheral CD4 counts (median 399 (333–901) cells/µL of blood) and decreased frequencies of CD4 T-cells in the gut mucosa relative to SIV-naïve controls (**Supplementary Figure 2**, **Supplementary Table 1**). Blood, peripheral lymph nodes (PLN), cerebrospinal fluid (CSF), and rectal samples (biopsy, cytobrush) were longitudinally collected according to **Figure 2A** for 4 weeks until the time of necropsy. In the SIV+ZIKV+ cohort, SIV viremia and peripheral CD4 counts remained stable post-ZIKV coinfection and there was no evidence of enhanced gut barrier dysfunction (**Supplementary Figures 2**, **6**). The most notable histologic findings were in Z16297, who had proliferative-occlusive pulmonary arteriopathy with thrombosis and infarction, which is a retroviral-strain-associated disease that was likely secondary to the SIV infection (**Supplementary Table 2**). Overall, these findings suggest that acute ZIKV co-infection does not have a significant effect on peripheral SIV viral replication or disease progression during the 4-week period examined.

### 3.4 SIV infection delays ZIKV viremia and increases ZIKV persistence in the gut mucosa

To assess the impact of the post-acute phase of SIV infection on ZIKV pathogenesis and tissue tropism, ZIKV burden was evaluated in longitudinal specimens and in tissues at necropsy by qRT-PCR. ZIKV RNA was not detected in one animal in the SIV+ZIKV+ cohort (Z14109) in any longitudinal sample tested nor in any necropsy tissue (**Supplementary Tables 7**, **8**); therefore, there was no evidence of productive ZIKV infection, and the animal was excluded from all subsequent post-ZIKV analysis. In the SIV-ZIKV+ cohort, plasma viremia peaked 2-4 days post infection (dpi) (median 3 dpi) and ZIKV was cleared in the plasma in most animals (6/7) by 7 dpi (**Figure 2B**). The mean viral kinetics in this cohort was consistent with our previous findings in ZIKV-infected PTM (30). In contrast, in SIV+ZIKV+ PTM, peak ZIKV viremia was variable (1-10 dpi; median 4 dpi) and virus was still present in most animals (4/7) by 7 dpi (**Figure 2B**). Accordingly, median plasma viral loads at 2 and 3 dpi trended to be higher or significantly higher (2 dpi, p = 0.108; 3 dpi, p = 0.050) in the naïve animals compared to SIV+ZIKV+ PTM and at 7 dpi trended higher (p = 0.054) in SIV+ZIKV+ PTM (**Supplementary Figure 7**). Together, these data indicate that peak ZIKV viremia in co-infected animals is heterogeneous, owing to inter-animal variability; however, there is a delay in initial ZIKV viremia and clearance in the periphery in SIV-infected pigtail macaques that is consistent with previous findings in rhesus macaques (34).

In pigtail macaques, we previously detected ZIKV viral RNA in the lymphoid and gastrointestinal tissues (30); therefore, we next examined if SIV infection altered ZIKV viral burden or persistence in tissues. In PLN, ZIKV RNA was detected in most animals (SIV- 5/7; SIV+ 4/5) at 7 dpi and in a proportion of animals (SIV- 2/7; SIV+ 2/6) at 21 dpi (**Figure 2B**). In rectal biopsy tissue, ZIKV RNA was detected in 4/7 of SIV- and 2/5 of SIV+ animals at 7 dpi, and at the time of necropsy (24-18 DPI) was detected in 1/7 of SIV- and half (3/6) of SIV+ animals at necropsy (**Figure 2B**; **Supplementary Figure 7**), suggesting ZIKV persistence in the rectum during SIV infection. In rectal cytobrushes, ZIKV RNA was predominantly detected in SIV- animals at 7 dpi (4/7 PTM) and was sporadically, but more consistently detected in SIV+ animals at 10 dpi (4/6 PTM), and the total viral burden in rectal cytobrushes trended (p = 0.171) to be higher during SIV+ZIKV+ co-infection (**Figure 2B**; **Supplementary Figure 7**). ZIKV RNA was not detected in CSF of any animal at any timepoint throughout the study (**Supplementary Table 7**) and corroborated our previous findings in PTM (30). Overall, these data suggest that ZIKV infectivity of the gut mucosa is more common and may be more persistent during post-acute SIV infection.

To evaluate the impact of SIV infection on ZIKV tropism and tissue persistence, we measured ZIKV viral burden in lymphoid, gastrointestinal (GI) mucosal, and neuronal tissues at necropsy (24-28 dpi). Consistent with our previous findings (30), ZIKV RNA was commonly detected in lymphoid tissues, variably in GI tissues, but not in brain tissue (including the brainstem, hippocampus, frontal lobe, parietal lobe, and occipital lobe), supporting the absence of neurotropism, as suggested by CSF analysis (**Figure 2C**, **Supplementary Table 8**). At necropsy, the total number of ZIKV positive tissues (p = 0.108) and overall viral burden (p = 0.171) trended higher in SIV+ZIKV+ animals compared to SIV-ZIKV+ animals (**Supplementary Figure 8**). Upon further investigation, we focused on ZIKV viral burden in gastrointestinal-associated tissues (e.g. GI mucosa and GI draining lymph nodes) and other lymphoid tissues. In both cohorts, ZIKV RNA was detected in at least one lymphoid tissue in most animals (5/6 SIV+ZIKV+; 4/7 SIV-ZIKV+) (**Figure 2C**) and ZIKV burden in individual lymphoid tissues or across all lymphoid tissues did not significantly differ between groups (**Figure 2D**; **Supplementary Figure 8**). In the GI-draining lymph nodes (mesenteric and iliac), ZIKV RNA was detected in both cohorts, but the individual tissue burden trended to be higher (p = 0.109) and total GI viral burden was significantly greater (p = 0.023) in the SIV+ZIKV+ group when compared to the SIV-ZIKV+ group (**Figures 2C, D**; **Supplementary Figure 8**). ZIKV RNA was detected in at least one GI tissue in most SIV+ PTMs (5/6) but only in a few SIV- PTM (2/7) (**Figure 2C**), reinforcing our previous observation that persistent ZIKV infection in the gut is uncommon in healthy PTM (30). Consistent with this, the median number of ZIKV-positive gastrointestinal tissues trended higher (p = 0.057) in SIV-infected animals and the total GI viral load also trended higher (p = 0.125) during SIV co-infection (**Supplementary Figure 8**). Moreover, a strong positive correlation (r = 0.7370, p = 0.0063) was observed between ZIKV burden in GI tissues and GI-draining lymph nodes (**Figure 2E**). In summary, while ZIKV distribution and persistence are similar across lymphoid tissues, ZIKV detection and viral burden are more common and persistent in the gut mucosa and GI-associated lymphoid tissues during post-acute SIV infection.

### 3.5 Delayed and dampened expansion of ZIKV cellular targets in blood corresponds with the recruitment of cellular targets to tissues during SIV-ZIKV co-infection

**Figure 3:**
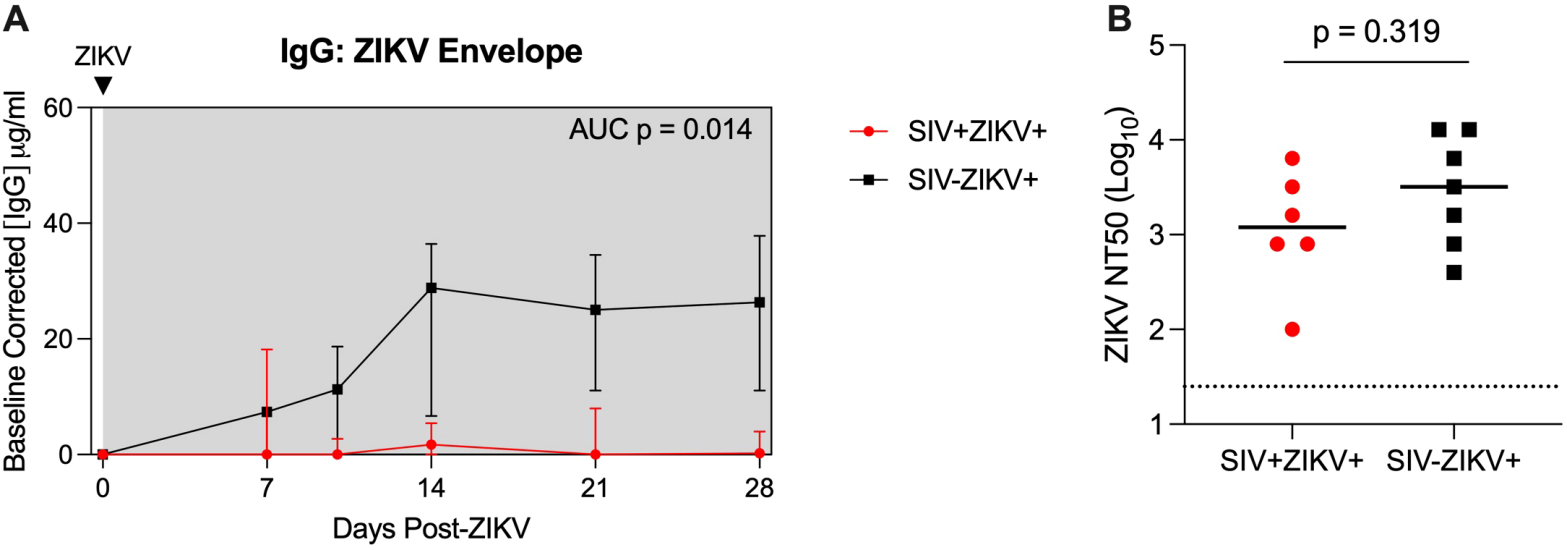
SIV infection may impair anti-ZIKV immunity. **(A)** Longitudinal plasma concentrations of anti-ZIKV envelope IgG as determined by ELISA. AUCs were calculated from day 10 to 28. Medians with interquartile ranges are displayed. **(B)** Zika virus neutralization antibody titers (NT50 values) evaluated at necropsy. The line represents the median and the dotted line represents is the limit of detection. **(A, B)** Mann-Whitney test comparison between groups.

As there was evidence for altered ZIKV pathogenesis in SIV-infected animals, we next evaluated whether this was associated with changes to ZIKV cellular targets or the immune response. Humoral responses are important for the control of ZIKV infection (51) and we measured anti-ZIKV envelope IgG responses in longitudinal plasma samples. SIV-ZIKV+ animals generated robust binding IgG antibodies that were detected at 7 dpi and peaked at 14 dpi, however these responses were lower overall in SIV+ZIKV+ animals (AUC, p = 0.014) (**Figure 3A**). Despite the difference in anti-ZIKV IgG between the two cohorts, similar levels of neutralizing antibodies (NAb) were generated against ZIKV at the time of necropsy (7/7 SIV-ZIKV+, 6/7 SIV+ZIKV+) (**Figure 3B**). Thus, neutralizing antibody production was equivalent in naïve and SIV+ZIKV+ animals, despite overall lower anti-ZIKV envelope IgG binding antibodies in the co-infected group.

**Figure 4:**
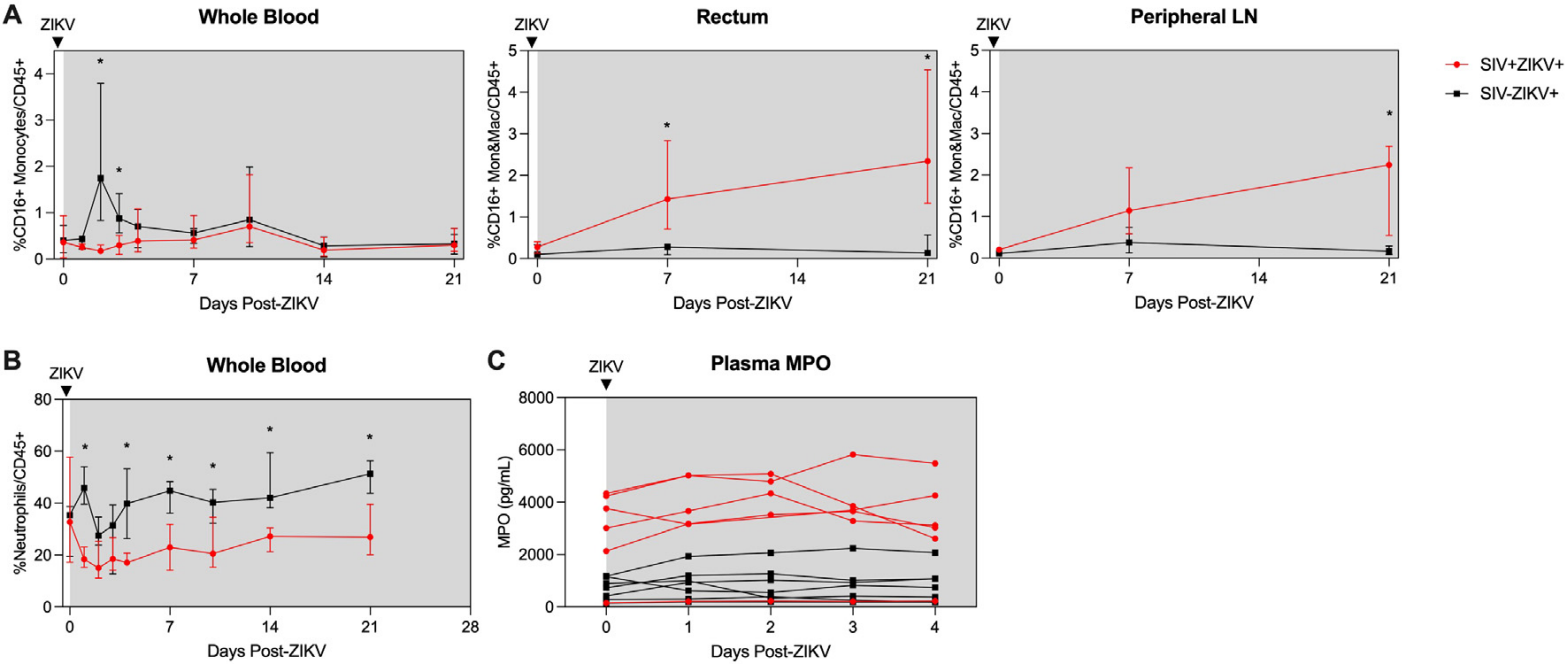
Post-ZIKV recruitment of CD16+ monocytes and macrophages is dampened in the periphery, but enhanced in tissues in SIV-infected macaques. **(A)** Frequency of CD16+CD14+ monocytes and macrophages (Mon&Mac) in blood (left panel), rectum (center panel), and peripheral lymph node (right panel) after ZIKV infection. **(B)** Frequency of neutrophils in blood after ZIKV infection. **(C)** Concentration of myeloperoxidase (MPO) in plasma as measured by ELISA. **(A-C)** Medians with interquartile ranges are shown. Mann-Whitney test between groups, p-values * ≤ 0.05.

Consistent with our previous findings (30), there is rapid and robust expansion of inflammatory CD16+ (non-classical and intermediate), CD16- (classical) monocytes, and DCs in the blood in the first few days after ZIKV infection (median peak 2 dpi), which corresponds to peak ZIKV viremia (**Figure 4A**; **Supplementary Figure 9**). In contrast, the expansion of CD16+ and CD16- monocytes was severely dampened and delayed during SIV infection (median peak 8.5 days) and corresponded with the delayed peak ZIKV viremia observed in these animals (**Figure 2B**). Cellular analysis in the tissues revealed that CD16- monocytes/macrophages were robustly and significantly recruited to the rectum and PLN in SIV- negative animals, whereas in contrast, inflammatory CD16+ monocytes/macrophages were recruited to the tissues during SIV+ZIKV+ co-infection (**Figure 4A**; **Supplementary Figure 9**). DC frequencies in the tissues were similar between the two groups (**Supplementary Figure 9**). AXL expression was not significantly changed on ZIKV cellular targets in whole blood, rectum and PLN in either group post-ZIKV infection (**Supplementary Figure 10**). These findings suggest that increased recruitment of inflammatory monocytes and macrophages to lymphoid and gastrointestinal tissues during SIV infection, which are also cellular targets of Zika virus infection, may contribute to ZIKV viral persistence at these sites.

Neutrophils are important for ZIKV dissemination and pathogenesis (30, 52, 53). Blood neutrophils declined during SIV+ZIKV+ co-infection starting at 1 dpi and were significantly lower in frequency in comparison to SIV-ZIKV+ animals through 21 dpi (**Figure 4B**). The frequencies of neutrophils in the rectum at 21 dpi were also lower in SIV+ZIKV+ compared to SIV-ZIKV+ animals, with no differences observed within the PLN (**Supplementary Figure 11**). To assess neutrophil function, we evaluated plasma concentrations of myeloperoxidase (MPO), a neutrophil granule and marker of inflammation, during the first 4 days of ZIKV infection. Interestingly, prior to ZIKV infection, elevated levels of MPO were detected in 5/6 SIV+ animals while lower concentrations of MPO were detected in 7/7 SIV- animals (**Figure 4C**). Post-ZIKV infection, concentrations of plasma MPO continued to be higher in SIV+ZIKV+ animals in comparison to SIV-ZIKV+ animals (**Figure 4C**). These data suggest that neutrophils are more inflammatory during SIV infection and impaired in their recruitment to the blood and to gastrointestinal tissues post-ZIKV.

### 3.6 SIV-ZIKV co-infection induces persistent innate immune activation

The inflammatory response to ZIKV in the plasma was evaluated using a multiplex immunoassay. In both groups, ZIKV induced pro-inflammatory responses, characterized by transient increases in interleukin-1 receptor agonist (IL-1RA), monocyte chemoattractant protein-1 (MCP-1), and vascular endothelial growth factor A (VEGF-A) (**Supplementary Figure 12**), with no major differences between groups. Although we found no evidence of ZIKV infection in neuronal tissue, markers of neuroinflammation were evaluated in longitudinal CSF specimens. IL-1RA, IL-6, IL-8, and VEGF-A were elevated in the CSF of several animals across groups at varying timepoints (**Supplementary Figure 12**). Transient increases in IL-8 were detected in the CSF of a few SIV-ZIKV+ animals after ZIKV infection, which is evidence of neuroinflammation. At necropsy, two animals in the SIV-ZIKV+ group (L07201 and Z08062) had mild, multifocal demyelination and axonal loss in the brainstem, which may be a result of neuroinflammation despite no evidence of ZIKV infection at this site (**Supplementary Tables 2**, **8**). CSF concentrations of sCD14, another marker of neuroinflammation, did not change with ZIKV infection in either group (**Supplementary Figure 6**). Overall, no significant differences between SIV+ZIKV+ vs SIV-ZIKV+ groups were found for any analytes detected in plasma or CSF during ZIKV infection, suggestive that SIV infection does not enhance ZIKV-induced systemic inflammation or neuroinflammation.

**Figure 5:**
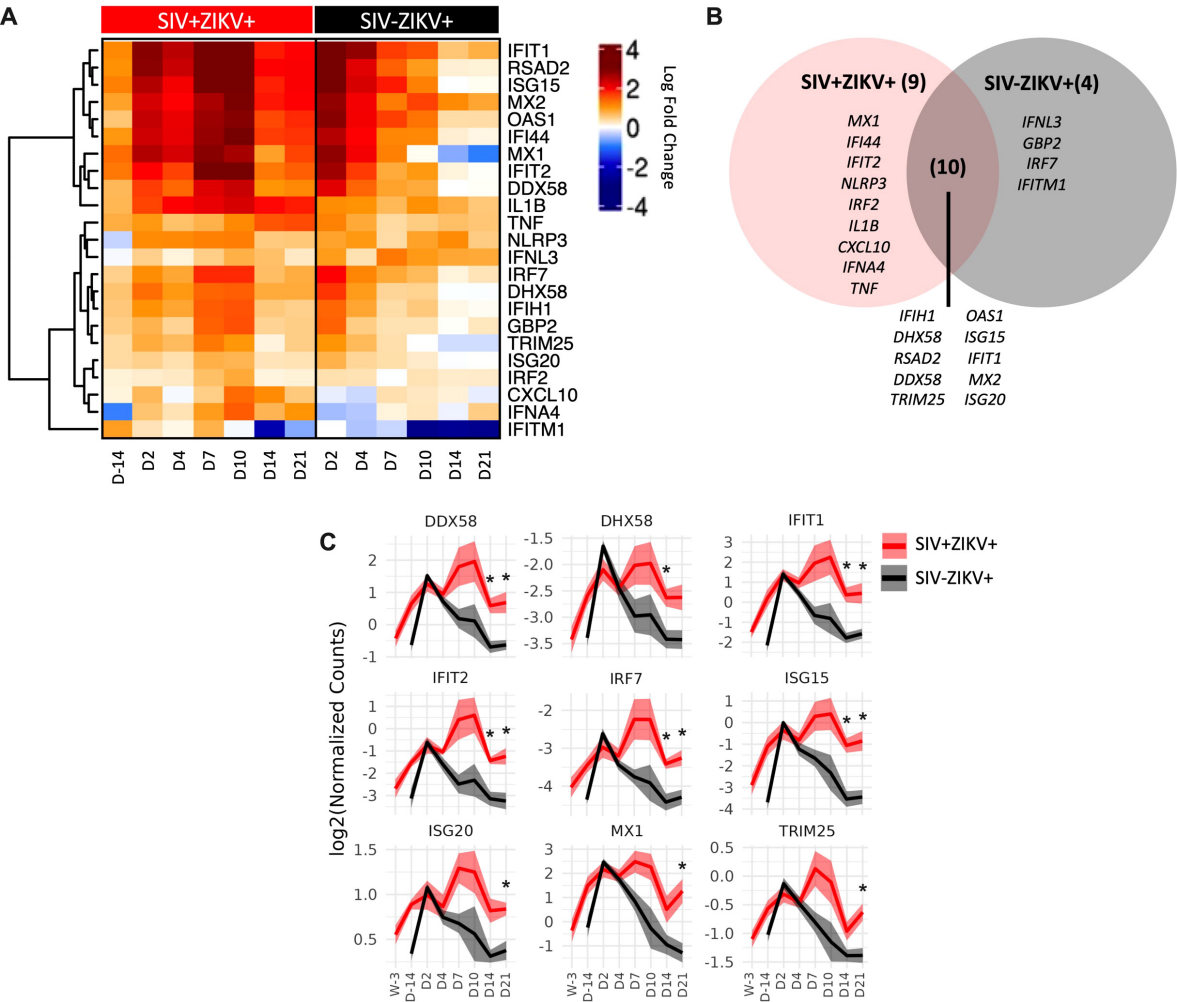
SIV-ZIKV co-infection induces persistent innate immune activation in PBMC. **(A)** Heatmap showing the Log2FC expression of 23 genes that were significantly different (*p*-value <0.01) in at least one timepoint and one group. Log2FC expression for the SIV+ZIKV+ group is relative to pre-SIV (Wk-3). Log2FC expression for the SIV-ZIKV+ group is relative to pre-ZIKV (D-14). Genes’ Log2FCs were clustered using Pearson and Ward.D2. **(B)** Venn diagram of shared and unique genes that were significantly upregulated post-ZIKV. **(C)** Line plots of select gene kinetics representing the mean of all SIV+ZIKV+ (red) or SIV-ZIKV+ (black) animals. The log2 normalized counts are plotted at each timepoint. The line represents the mean and the standard error is shown as the confidence interval around the mean. p-values * <0.01 indicates a significant difference between SIV+ZIKV+ and or SIV-ZIKV+ at a specified timepoint.

To further examine SIV effects on immune responses to ZIKV, we performed targeted gene expression analysis on longitudinal PBMC specimens collected post-ZIKV infection using a custom-built NanoString Code Set for interrogating 64 genes marking innate immune activation, inflammatory and antiviral responses. Changes in gene expression were determined at each timepoint post-challenge in comparison to uninfected timepoints. Gene expression was not significantly changed 7 weeks post-SIV infection relative to naïve PBMC. This indicated that while SIV infection stimulated gene expression, the changes just prior to ZIKV challenge were not significantly different from baseline levels. Following ZIKV challenge, a total of 23 genes were significantly differentially expressed (22 upregulated and 1 downregulated), 14 genes in SIV-ZIKV+ (13 up and 1 down), 19 genes in SIV+ZIKV+ (19 up), and 10 genes in both groups (10 up) (**Figure 5B**; **Supplementary Table 5**). ZIKV induced robust innate immune gene activation in SIV-ZIKV+ animals during acute infection (2-4 dpi), with the expression of most genes returning to baseline by 7 dpi (**Figure 5A**). The kinetics of the gene expression mirrors peak ZIKV viremia (median 3 dpi) and time to viral clearance (median 7 dpi). While these innate immune genes were also strongly upregulated in SIV+ZIKV+ co-infected animals during acute infection (2-4 dpi), in contrast to the SIV-ZIKV+ group, the gene signature was maintained in SIV+ZIKV+ animals throughout infection and remained highly expressed 7-21 dpi. The kinetics of gene expression during SIV+ZIKV+ co-infection corresponds to the shift in peak ZIKV viremia (median 4 dpi) and viral clearance (median 10 dpi). We also found that the expression of 10 genes were significantly different between SIV+ZIKV+ and SIV-ZIKV+ animals, primarily at 14 and 21 dpi, and predominantly consisted of genes associated with type I IFN signaling and ZIKV viral control (*ISG15, IFIT1, MX1, ISG20, IRF7)* (**Figure 5C**, **Supplementary Table 6**). These data demonstrate that in the blood, SIV-ZIKV co-infection leads to persistent upregulation of genes associated with inflammation and innate immune activation. This persistent and hyperactivated innate immune state in response to ZIKV co-infection likely contributes to the impaired peripheral ZIKV clearance and its persistence in tissues.

### 3.7 Immune factors associated with ZIKV persistence in gastrointestinal tissues

**Figure 6:**
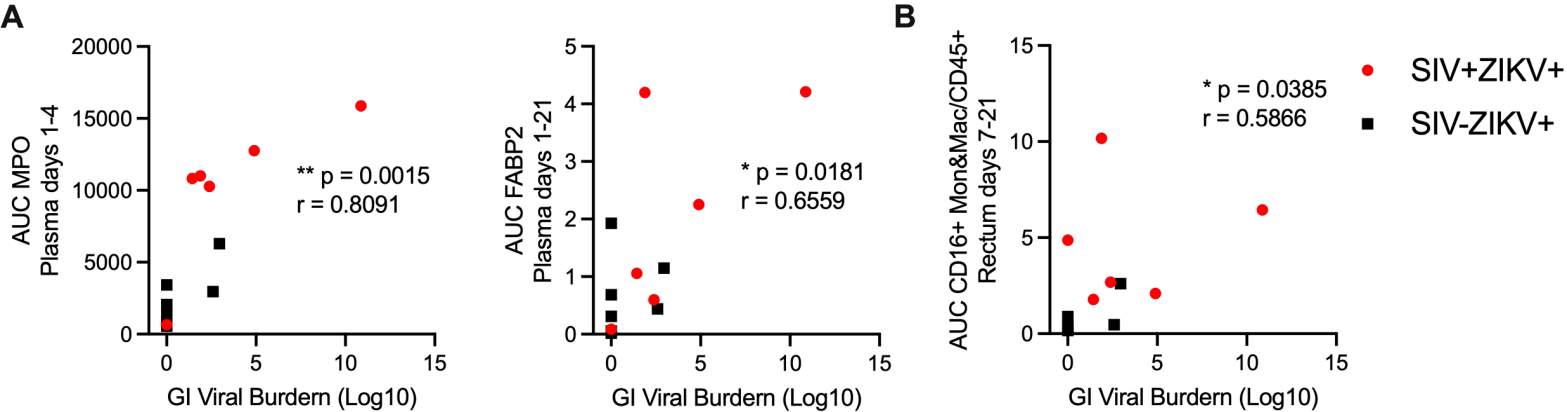
Immune responses and innate gene signature associated with persistent ZIKV RNA in the gastrointestinal tract. Scatter plot and Spearman’s correlation analysis of the relationship between the gut ZIKV viral burden at necropsy and **(A)** plasma MPO (AUC, days 1-4) (left panel), plasma FABP2 (AUC, days 1-21) (right panel) or **(B)** CD16+ monocytes and macrophages (Mon&Mac) in rectum (AUC, days 7-21). **(A, B)** Spearman’s correlation test p-values are indicated as follows: *p ≤ 0.05,**p ≤ 0.01.

Studies in NHP and human have revealed that ZIKV can persist in bodily fluid and tissues, including semen, CSF, lymphoid and gastrointestinal tissues for several months after infection; however, the mechanisms contributing to persistence are poorly understood (14, 33). In the pigtail macaque model, we previously found ZIKV persistence in lymphoid, but not in gastrointestinal, tissues (30). As several of our animals had ZIKV RNA present in gastrointestinal tissues at necropsy and was more common in SIV+ (5/6) versus SIV- (2/7) animals, we next examined the immune responses associated with ZIKV gut persistence, irrespective of SIV status. We performed correlational analysis between immune responses in the blood or rectum to levels of GI ZIKV burden, a summation of the gut viral load in each tissue at necropsy (**Supplementary Table 9**). Among these, higher concentrations of plasma MPO (r = 0.8091, p = 0.0015), indicative of an inflammatory neutrophil response, higher concentrations of FABP2 (r = 0.6559, p = 0.0181), a marker of gut barrier dysfunction, and greater infiltration of inflammatory CD16+ monocytes and macrophages into the rectum (r = 0.5866 p = 0.0385) all positively correlated with higher ZIKV gut burden at necropsy (**Figures 6A, B**).

## 4 Discussion

In this study, we investigated whether untreated HIV-induced immunosuppression impacts ZIKV pathogenesis using *in vitro* and *in vivo* SIV-ZIKV co-infection models. We found that peripheral ZIKV cellular targets, including CD16+ monocytes, increase during acute SIV infection but contract to baseline levels during the post-acute stage. Although AXL is a key receptor of ZIKV infection, we found no change in AXL expression on monocytes in whole blood or tissues of SIV+ animals after ZIKV challenge, suggesting that enhanced ZIKV persistence during SIV infection is not due to increased receptor engagement. Interestingly, PBMC from acutely SIV infected NHP exhibited an anti-viral gene expression profile that renders them refractory to ZIKV co-infection *in vitro*. *In vivo*, we demonstrated that SIV infection promotes ZIKV persistence in gastrointestinal mucosal and lymphoid tissues. SIV infection also modulated both the innate and adaptive immune responses to ZIKV, creating a hyper-inflammatory state that lasted for weeks after the post-acute stage of ZIKV infection, which could further contribute to prolonged viremia and impaired viral clearance from the tissues, particularly in the gastrointestinal tract. These findings suggest that PLWH or other immunocompromised individuals co-infected with ZIKV could experience prolonged periods of ZIKV transmission.

The clinical impact of HIV co-infection on flavivirus infection, including ZIKV, remains unclear. Studies on DENV-HIV co-infection demonstrate conflicting results, with some indicating less severe disease and others reporting more severe outcomes (54–56). ZIKV infection during pregnancy in women living with HIV (WLWH) is of great concern. One study reported 12% of ZIKV-exposed infants from WLWH had CNS abnormalities (57), compared to 5-8% in infants from HIV-negative women (58), suggesting a higher risk of neuropathologies in HIV-ZIKV co-exposed infants. However, these studies are limited by small sample sizes, inconsistent incorporation of ART and HIV disease status, and a focus primarily on blood-based measures. NHP studies, including those using SIV or SHIV models, have helped address some of these gaps. For instance, Rosinski, et al., reported similar pregnancy loss rates in SIV-naïve and SIV infected/ART-treated macaques (59), although the impact of untreated SIV on fetal loss remains unexplored. Two studies have investigated ZIKV in non-pregnant rhesus macaques with untreated SIV/SHIV infection (34, 60). Bidokhti et al., reported no significant differences in ZIKV viremia between SIV/SHIV infected and uninfected macaques; however, interpretations from this study are limited by low animal numbers and a lack of contemporaneous controls (60). In contrast, Vinton et al., observed delayed peak ZIKV viremia and slower viral clearance in SIV-infected rhesus (34), consistent with our findings in pigtail macaques. They also detected higher levels of ZIKV RNA in lymph nodes of SIV+ compared to SIV- rhesus macaques at 27 dpi (34). We also observed ZIKV persistence in lymph nodes in both SIV+ and SIV- pigtail macaques, but we found no significant differences in viral burden between the groups. However, we did find that viral burden in gut-draining lymph nodes was significantly higher in SIV+ versus SIV- animals. These differences may be due, in part, to the sensitivities of the methods used– fluorescence *in situ* hybridization in Vinton et al., versus qRT-PCR in ours—or may reflect species-specific variations.

Long-term ZIKV persistence in lymph node following viral clearance in the periphery is correlated with induction of IFNα and acute inflammatory signaling pathways in PBMC of rhesus macaques (33). We find *CXCL10*, *IFI44*, and *IL1B* genes associated with these pathways have increased and sustained expression in PBMC of SIV+ZIKV+ animals. ZIKV replication in the periphery is restricted by early innate immune responses; however, ZIKV antagonism of type I IFN defenses via STAT2 degradation leads to upregulation of proinflammatory signaling pathways. Consequently, viral persistence is established by antagonizing cytoxic T lymphocyte responses and creating a specific ISG response in bystander cells within tissues that could serve to limit ZIKV tissue spread and systemic infection. Although, ZIKV RNA persistence in the gastrointestinal tract is previously reported in naïve rhesus macaques (33), it has not been detected in pigtail macaques (30), and the mechanisms contributing to gut persistence are unknown. Our study uniquely shows that SIV infection promotes ZIKV persistence in gastrointestinal tissues, a compartment not previously reported in SIV-ZIKV co-infection studies. We observed that RNA persistence in the GI tract correlates to viral burden in the GI-draining lymph nodes, suggesting that the virus may ‘cross-seed’ or repopulate between these anatomical sites. Collectively, our findings, along with those of Vinton et. al., suggest that untreated acute SIV infection alters ZIKV viral kinetics and promotes ZIKV persistence or viral burden in tissues. However, whether tissue inflammation plays a similar role remains to be explored. These results imply that immunocompromised individuals, including PLWH, may experience prolonged ZIKV transmission and altered disease pathogenesis, making them primary candidates for ZIKV vaccines.

ZIKV and West Nile Virus (WNV) can invade the central nervous system, yet the mechanisms behind neuropathogenesis remain poorly understood (3, 4). Detection of ZIKV RNA in the CSF or brain occurs infrequently, but consistently in rhesus and cynomolgous macaques (26, 31, 33, 61, 62); however, ZIKV RNA is neither detected in CSF nor CNS of adult pigtail macaques (30). Neuroinflammation has been reported in ZIKV infected NHP, even when ZIKV RNA is undetectable in the CSF (33, 63–65). Previous studies have shown increased inflammatory markers, including IL-15, MCP-1, G-CSF, and CXCL12, in the CSF of ZIKV infected rhesus (63, 64). In our study, we also observed elevated levels of IL-1RA, IL-6, and IL-8 in the CSF, but these responses were highly variable between animals. The differences in severity of ZIKV neuroinvasion and cytokine milieus between our study in pigtail macaques and those in rhesus macaques could be due to variations in assay sensitivity, or they could reflect true species differences in neuroinflammatory responses. In PLWH, increased WNV neuroinvasion is reported (66–68), suggesting that HIV-ZIKV co-infection might similarly increase the risk of ZIKV targeting to the CNS and result in higher rates of neurological pathologies. While our study showed no evidence that SIV infection enhances ZIKV neuroinvasion or neuroinflammation, future efforts in the NHP HIV/AIDS model should investigate flavivirus pathogenesis in other NHP species and in the context of antiviral therapy to fully assess the risk of neuroinvasion and the duration of ZIKV persistence in tissues.

Many promising ZIKV vaccine candidates in the pre-clinical pipeline aim to induce antibody and T-cell responses for protection (69). Studies in NHP have shown that CD8 T-cells are not required for protection against primary or secondary ZIKV infections (31, 70). Additionally, research in NHP has demonstrated that CD4+ T-cell depletion impairs the generation of anti-flavivirus (DENV or ZIKV) humoral responses, which in turn shapes the quality of responses to a tertiary flavivirus exposure (71). Our study aligns with these findings, showing that SIV infection, which causes CD4+ T-cell immunodeficiency, impairs the development of adaptive immunity to ZIKV. Further research is needed to elucidate whether immune responses generated during CD4 immunosuppression can still protect against homologous and/or heterologous ZIKV re-exposure. This information will help identify at-risk populations and guide the development of effective vaccines for individuals with various states of immunosuppression, including the elderly, pregnant women, and those with compromised immune systems. Additionally, SIV-infected NHP serve as an important model for assessing the immunogenicity and effectiveness of new vaccine platforms, as well as for testing adjuvant-vaccine combinations aimed at boosting immunity in immunocompromised individuals.

The innate immune response is the body’s primary defense against flavivirus infection, but if dysregulated, it can exacerbate viral pathogenesis. This dichotomous role is further complicated during flavivirus infection, as innate immune cells—such as monocytes and dendritic cells—are direct targets of ZIKV, WNV, and DENV infection. We found *in vitro* that PBMC from SIV-infected animals were less permissive to ZIKV infection and that *in vivo*, ZIKV viremia was delayed. Initially, cells from immunosuppressed individuals may be less permissive to ZIKV infection due to an antiviral state, which could slow the establishment of ZIKV infection. However, once ZIKV infection is established, the ability to recruit immune cells crucial for clearing the virus is impaired and dysregulated during immunosuppression, promoting ZIKV dissemination and persistence, particularly within the gastrointestinal tract and GI-associated lymphoid tissues. While ZIKV persistence in the CSF and lymph nodes has been linked to the upregulation of mTOR, proinflammatory, and anti-apoptotic signaling pathways (33), the mechanisms driving persistence in the gut mucosa are unknown. Previously, we reported that the infiltration of neutrophils and CD16- monocytes/macrophages into tissues helps limit ZIKV replication in mucosal tissues (30). Here, we found that increased recruitment of inflammatory CD16+ monocytes/macrophages to tissues, higher peripheral inflammatory neutrophils, and compromised GI integrity were associated with ZIKV RNA persistence in the gut mucosa, but other unexplored mechanisms may also contribute to persistence. Future studies at the single-cell level, including single cell RNA-seq, could identify ZIKV-infected cell types in the gut and the cell-specific transcriptional response associated with persistent ZIKV infection. Prolonged innate immune activation is a hallmark of chronic infections, including HIV, but can also occur following acute viral infections. For example, in SARS-CoV-2 infection, the virus can persist in the tissues or lead to post-acute sequelae of COVID-19 (PASC) (72). PASC is associated with persistent immune activation and PLWH are at higher risk (73). Furthermore, SARS-CoV-2 persistence occurs in people with untreated or advanced HIV (74, 75). This suggests that persistent innate immune activation can be a major driver in promoting viral persistence. Our study further supports this idea, suggesting that ongoing peripheral innate immune activation could contribute to ZIKV persistence in tissues. Thus, immunocompromised individuals, including PLWH, may experience prolonged ZIKV transmission and altered disease pathogenesis, making them primary candidates for ZIKV vaccines.

## Data Availability

The original contributions presented in the study are included in the article/**Supplementary Material**, further inquiries can be directed to the corresponding author. The R codes applied to these analyses can be accessed at https://github.com/galelab/OConnor_SIV-ZIKV_coinfection.
