# Supplementary material for: Persistent innate immune dysfunction and ZIKV replication in the gastrointestinal tract during SIV infection in pigtail macaques
Source: Front Immunol. 2025 Mar 4;16:1535807. doi: 10.3389/fimmu.2025.1535807 (PMC11913663; doi:10.3389/fimmu.2025.1535807)
Supplement: Supplementary file 1 [file DataSheet1.docx]

Supplementary Material

# Supplementary Tables and Figures

## Supplementary Tables

**Supplementary Table 1**. Animal Characteristics

**Supplementary Table 2**. Histology

**Supplementary Table 3**. Antibodies

**Supplementary Table 4**. PBMC gene expression post-SIV infection

**Supplementary Table 5**. PBMC gene expression post-ZIKV infection

**Supplementary Table 6**. PBMC gene expression comparison of SIV+ZIKV+ and SIV-ZIKV+ groups

**Supplementary Table 7**. ZIKV RNA analysis in longitudinal specimen

**Supplementary Table 8**. ZIKV RNA analysis in necropsy tissue specimen

**Supplementary Table 9**. Correlation analysis between gut viral burden at necropsy and immune responses

## Supplementary Figures

**Supplementary Figure 1**. Gating strategies used for immune analyses.

**Supplementary Figure 2**. Quantification of ZIKV RNA and viral titer in PBMC from SIV-infected PTM.

**Supplementary Figure 3**. Changes in expression of cytokines and chemokines post-*in vitro* ZIKV infection.

**Supplementary Figure 4**. AXL expression on ZIKV cellular targets.

**Supplementary Figure 5**. Gut barrier disfunction and neuroinflammation does not increase during SIV-ZIKV coinfection.

**Supplementary Figure 6**. Kinetics of Zika viral burden in tissues.

**Supplementary Figure 7**. ZIKV RNA detection in tissues at necropsy.

**Supplementary Figure 8**. Frequency of monocytes and dendritic cells in blood and tissues.

**Supplementary Figure 9**. Changes in cellular AXL expression post-ZIKV infection.

**Supplementary Figure 10**. Changes in neutrophils within tissues.

**Supplementary Figure 11**. Changes in *ex vivo* expression of cytokines and chemokines post-ZIKV infection

**Supplementary Figure 1.**

**Supplementary Figure 1. Gating strategies used for immune analyses.** Representative gating strategy in blood to identify innate immune cells. Background gating on FSC singlets, then CD45+ cells, live cells, and cells according FSC-A and SSC-A profiles were performed on all cell types prior to identification of immune cell subsets. T-cells were identified as CD3+ and then divided into CD4+ and CD8+ subsets. Monocytes and dendritic cells (DCs) were first gated as CD3-, HLA-DR+ and then divided into CD14±CD16± for monocytes and CD14- for DCs. Monocytes (Mon) subsets were gated into classical (CD14+CD16-), intermediate (Int) (CD14+CD16+), and non-classical (Non-Class) subsets (CD14intCD16+) and and dendritic cells were gated into pDCs (CD123+CD11c-) and mDCs (CD123- CD11c+). CD16+ monocytes amd DCs were identified using Boolean gating. Neutrophils were identified as CD3-, CD11b+CD14+ and then selected for high SSC.

**Supplementary Figure 2.**

**Supplementary Figure 2.** **SIV-infected animals are immunosuppressed at the time of ZIKV co-infection.** (**A**) Plasma levels of Log10 SIV viral RNA levels were measured by RT-PCR. (**B**) Peripheral blood CD3+CD4+ T-cell counts were quantified from the complete blood count (CBC) following flow cytometry analysis. Frequency of CD3+CD4+ T-cells in the (**C**) peripheral lymph node (PLN) and (**D**) rectum as determined by flow cytometry. (**B-D**) Medians with interquartile ranges are shown. Mann-Whitney test between group, p-values * ≤ 0.05.

**Supplementary Figure 3.**

**Supplementary Figure 3. Quantification of ZIKV RNA and viral titer in PBMC from SIV-infected PTM.** Peripheral blood mononuclear cells (PBMC) were isolated from pigtail macaques prior to and at 2 and 6 weeks post-SIV infection and infected *in vitro* with ZIKV Brazil 2015 at MOI of 2. Cells and culture supernatants were harvested at 4, 24, and 48 hours post infection. (**A**) ZIKV RNA in PBMC determined by qRT-PCR assay. (**B**) Viral titers from PBMC culture supernatants determined by plaque assay. Means with standard deviations of 1-2 biological replicates are shown.

**Supplementary Figure 4.**

**
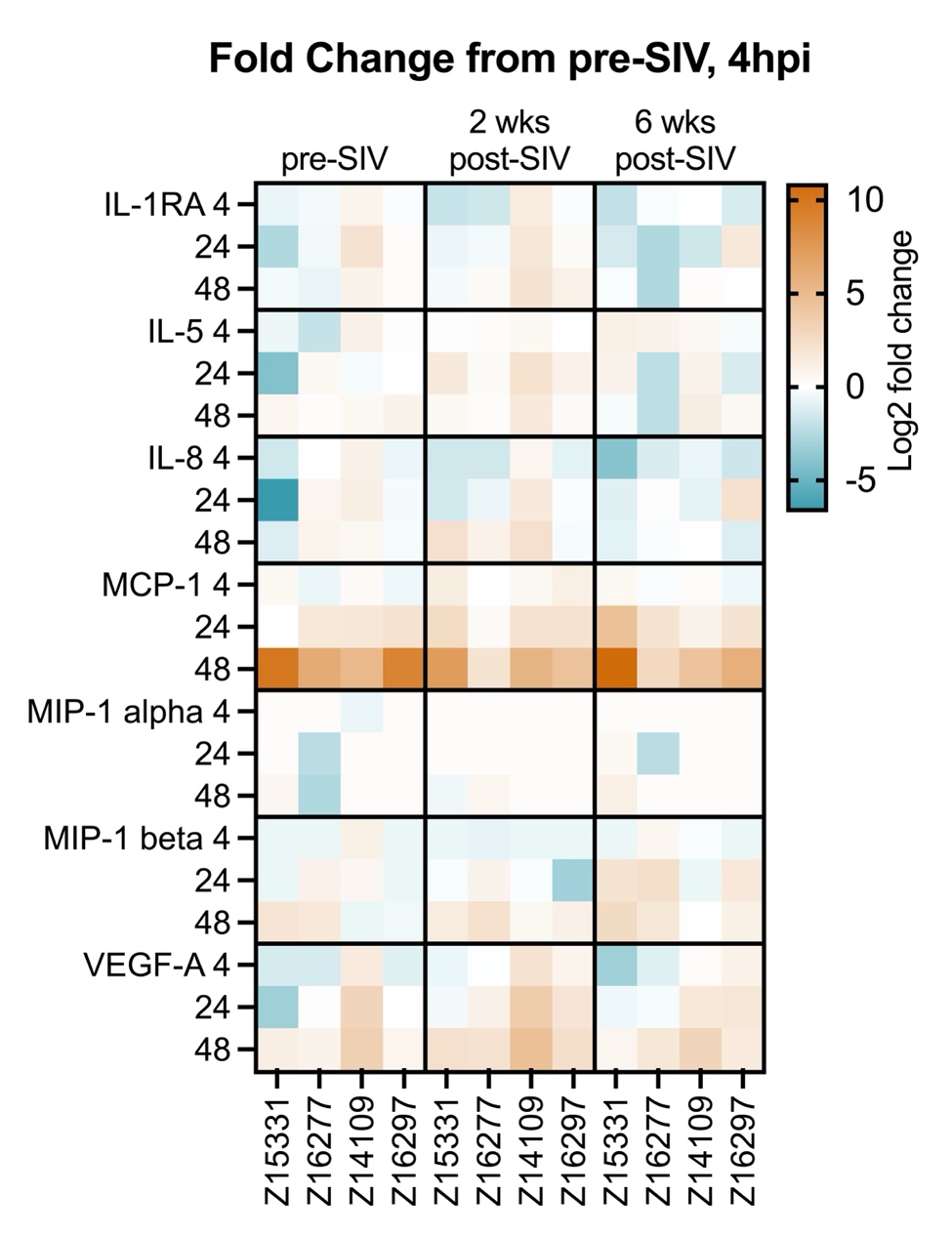
**

**Supplementary Figure 4. Changes in expression of cytokines and chemokines post-*in vitro* ZIKV infection.** Concentrations of cytokines and chemokines were determined by multiplex immunoassay in cell culture supernatants at 4, 24, and 48 hours post ZIKV infection. Log2 fold-change values from average 4 hpi pre-SIV baseline levels are shown.

**Supplementary Figure 5.**

**Supplementary Figure 5. AXL expression on ZIKV cellular targets.** Frequency AXL-expressing of CD16+CD14+ monocytes and macrophages (left panel) and dendritic cells (right panel) in blood from uninfected and SIV-infected pigtail macaques. Wilcoxon matched-pairs signed rank test, p-values ≤ 0.05 considered significant.

**Supplementary Figure 6.**

**Supplementary Figure 6. Gut barrier disfunction and neuroinflammation does not increase during SIV-ZIKV coinfection.** (**A**) Concentrations of soluble CD14 (sCD14) (top panel), fatty acid binding protein 2 (FABP2) (middle panel), and LPS binding protein (LBP) (bottom panels) in plasma (n=7/group) and (**B**) sCD14 levels in CSF (n=4/group) as determined by ELISA. No significant differences were found between groups using a Mann-Whitney test.

**Supplementary Figure 7.**

**Supplementary Figure 7. Kinetics of Zika viral burden in tissues.** Quantitative real-time PCR (qRT-PCR) for ZIKV RNA in longitudinal samples from (**A**) plasma, (**B**) rectal biopsies, (**C**) peripheral lymph node (PLN), and (**D**) rectal cytobrush. AUC analysis is from day 0 to 21. Mann-Whitney test of AUC between groups, p-values ≤ 0.05 are considered significant.

**Supplementary Figure 8.**

**Supplementary Figure 8. ZIKV RNA detection in tissues at necropsy.** ZIKV RNA was quantified in necropsy tissue by qRT-PCR. (**A**) Number of ZIKV RNA+ tissues (left panel) and total viral burden (right panel) in all tissues, (**B**) total viral burden of individual ZIKV RNA+ tissues within other lymphoid, GI-draining lymph node (LN) and GI tissues, and (**C**) number of ZIKV RNA+ tissues in gastrointestinal (GI) tissues. Each point represents an individual animal and/or tissue and medians are shown. Mann-Whitney test between groups, p-values ≤ 0.1 are considered trending.

**Supplementary Figure 9.**

**Supplementary Figure 9. Frequency of monocytes and dendritic cells in blood and tissues**. Frequency of (**A**) CD16-CD14+ monocytes and macrophages and (**B**) dendritic cells in blood (left panels), rectum (center panels), and peripheral lymph node (right panels) after ZIKV infection. Medians with interquartile ranges are shown. Mann-Whitney test between group, p-values * ≤ 0.05.

**Supplementary Figure 10.**

**Supplementary Figure 10. Changes in cellular AXL expression post-ZIKV infection.** Frequency of AXL expression on (**A**) CD16+ monocytes and (**B**) dendritic cells in blood (left panels), rectum (center panels), and peripheral lymph node (right panels) after ZIKV infection. No significant differences were found between groups using a Mann-Whitney test.

**Supplementary Figure 11.**

**Supplementary Figure 11. Changes in neutrophils within tissues.** Frequency of neutrophils in the rectum (left panel) and peripheral lymph node (right panel) after ZIKV infection. Medians with interquartile ranges are shown. Mann-Whitney test between groups, p-values * ≤ 0.05.

**Supplementary Figure 12.**


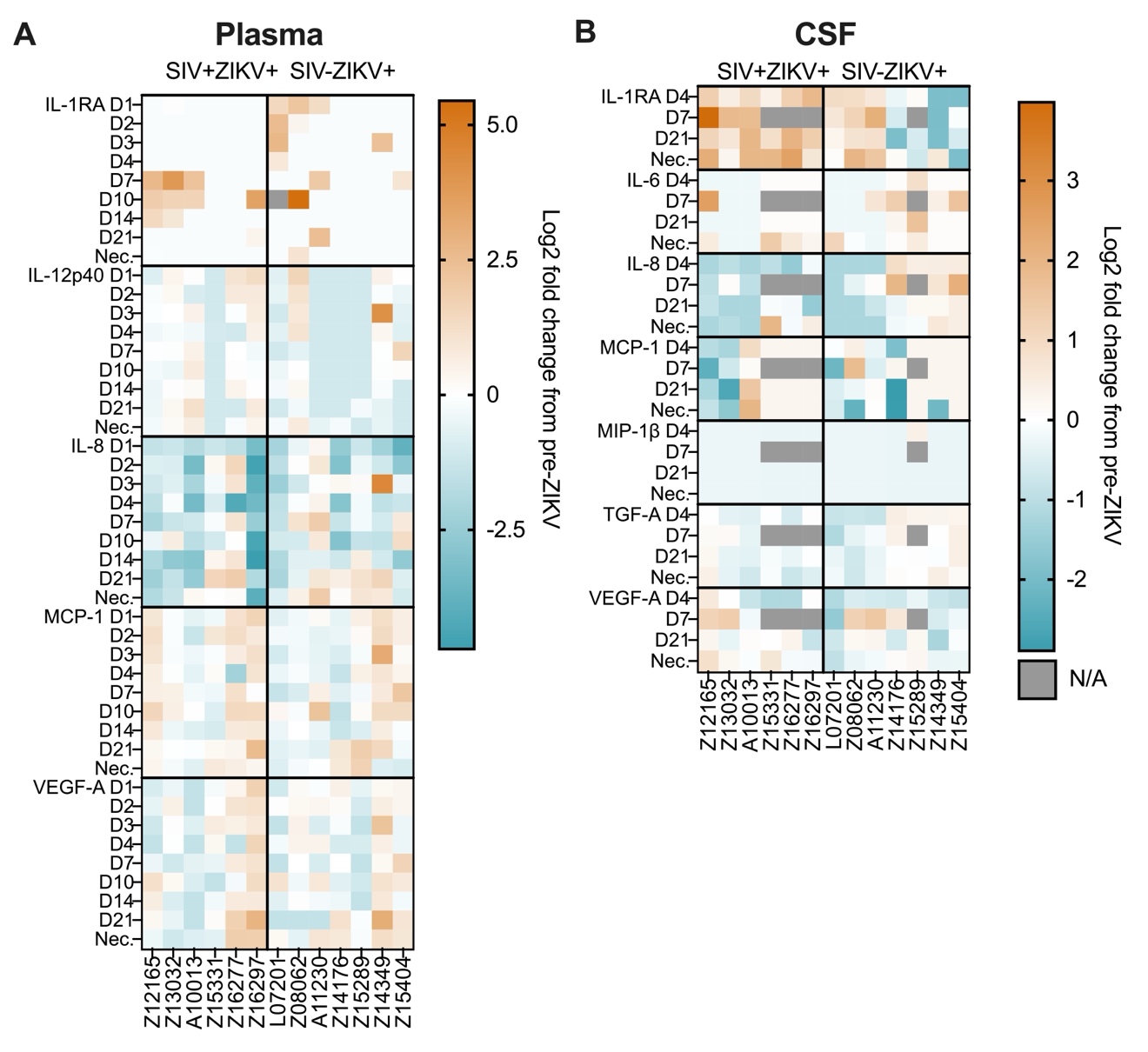


**Supplementary Figure 12. Changes in *ex vivo* expression of cytokines and chemokines post-ZIKV infection.** Concentrations of cytokines and chemokines were determined by multiplex immunoassay in (**A**) plasma and (**B**) CSF at the indicated days post-ZIKV infection. Log2 fold-change values from pre-ZIKV baseline levels are shown. N/A, not applicable; Nec., necropsy; D, day
